# Factors affecting the utilization of antenatal care among married women of reproductive age in the rural Thatta, Pakistan: findings from a community-based case-control study

**DOI:** 10.1186/s12884-020-03009-4

**Published:** 2020-06-10

**Authors:** Sumera Aziz Ali, Savera Aziz Ali, Anam Feroz, Sarah Saleem, Zafar Fatmai, Muhammad Masood Kadir

**Affiliations:** 1 Department of Epidemiology, Columbia University, New York, USA; 2 Department of Nursing, University of Alberta, Edmonton, Canada; 3 Department of Community Health Sciences, Aga Khan University Hospital, Karachi, Pakistan

**Keywords:** Road network distance, Antenatal care utilization, Rural Pakistan, Married women

## Abstract

**Background:**

There are differences in antenatal care (ANC) utilization between urban and rural areas of Pakistan. Although multiple factors have been studied affecting the utilization of general health care services, the effect of road network distance particularly on the utilization of ANC has not been assessed. Therefore, this study aimed to determine the association between road network distance from a health care facility and utilization of the ANC among women of reproductive age in Thatta Pakistan.

**Methods:**

A community-based case-control study was conducted in district Thatta, Pakistan. Women who did not utilize ANC services during their last pregnancy were considered as cases, while controls were the women who utilized ANC services during their last pregnancy. Questions related to socio-demographic, access-related factors and utilization of ANC were asked from women. Road network distance was calculated from the women’s home to the health care facility providing ANC services. Logistic regression analysis was performed.

**Results:**

A total of 380 participants were interviewed in this study. Participants’ mean age and parity were 28 years (SD 5.65), and 3.5 (SD 2.6) respectively. The multivariate analysis showed that women living at a shorter distance of less than 5 km were 1.21 times likely to utilize ANC services [Adjusted OR. 1.21; 95% CI (0.49–2.99)]. Moreover, nulliparous women were 4.10 times likely to utilize antenatal care [Adjusted OR. 4.10; 95% CI (1.10–15.26)]. Similarly, women who had knowledge of antennal care were 6.60 times likely to utilize ANC services [Adjusted OR. 6.60; 95% CI (3.33–13.05)]. Women having electricity in their households were 3.15 times likely to utilize the ANC services [Adjusted OR. 3.11 95% CI (1.51–6.41)]. Women, living in well-constructed (Pakka) houses were 2.58 times likely to utilize the ANC services [Adjusted O.R: 2.58; 95% CI (1.15–5.82)].

**Conclusion:**

Road network distance has no measurable impact on ANC utilization among married women in Thatta district, Pakistan. Nulliparous women having knowledge of ANC living in well-constructed houses equipped with electricity were found to be utilizers of ANC services. It is recommended that awareness and health education sessions should be arranged for pregnant women in rural Pakistan.

## Plain English language summary

A pregnant woman needs to get antenatal care on time as it is a key element to improve maternal and newborn health. Despite the benefits of antenatal care, the utilization of antennal care is not up to mark and there is a vast difference in antenatal care utilization between the rural and urban settings of Pakistan. There is a dearth of research studying the effect of road network distance on the utilization of antenatal care services mainly in the rural area of Pakistan. Therefore, there was a need to carry out research to assess the association between road network distance and the utilization of ANC among women. Hence this study was conducted to determine the association between road network distance from a health care facility and utilization of the ANC among women of reproductive age in Thatta Pakistan from October 2014 to December 2015. Road network distance from health care facilities did not impact the utilization of ANC services among women in Thatta district. However, it was found that women having one child, living in well-constructed houses, well furnished with electricity and who had knowledge of antenatal care were found to utilize the antenatal care services in the study area as compared to their counterparts. Hence women should be given more knowledge and awareness by conducting campaigns and health education sessions to improve antenatal care utilization. Moreover, socioeconomic status also needs to be improved to enable women utilizing ANC in rural areas of Pakistan. More studies are required in the future to study other factors at the individual, household, community, and facility-level to understand the determinants of utilization of antenatal care services holistically.

## Background

Globally thousands of women and infants are at the risk of dying due to complications related to pregnancy and delivery of the baby. For instance, approximately, 303, 000 women died and 2.6 million stillbirths occurred in 2015 due to problems associated with pregnancy and labor [1, 2]. Although considerable progress has been made over the last two decades still there is a need to improve access to quality care during pregnancy, labor, and delivery to reduce preventable deaths among women [3]. Care during pregnancy or antenatal care (ANC) is defined as the “care provided by trained health-care professionals to pregnant women and adolescent girls to ensure the best health conditions for both mother and baby during pregnancy” [4].

Furthermore, the World Health Organization (WHO) has advocated various approaches to prevent pregnancy-related complications. For instance, the FANC (focused antenatal care) model of WHO emphasizes that a pregnant woman should have comprehensive ANC visits, and be screened and treated for anemia, malaria, HIV/AIDS, and also be immunized against tetanus [5]. Moreover, recently WHO has recommended new guidelines related to ANC including numerous other changes to the FANC model. According to these new guidelines, a pregnant woman should have at least eight ANC visits, starting the first visit at 12 weeks’ of gestational age (GA), followed by visiting skilled health care provider at 20, 26, 30, 34, 36, 38 and 40 weeks’ of GA [6]. Moreover, these new guidelines highlight that a woman’s ‘contact’ with her provider should be beyond a simple ‘visit’. This ‘contact’ between a pregnant woman and a health care provider should be an opportunity for good quality care during pregnancy [7]. These new guidelines aim to ensure both a healthy pregnancy and a successful transition to labor and delivery [6]. In addition, these recommendations are more comprehensive focusing not only on maternal and fetal screening but also on counseling, maternal nutrition, prevention‚ and treatment of common ailments, providing support to women at risk of facing intimate partner violence and also on preventative measures for certain locales such as malaria and/or HIV endemic areas [4, 6].

Thus, screening during ANC provides an opportunity to assess the pregnancy risk, treating antenatal problems, providing health education‚ and awareness to the pregnant women, giving timely treatment to reduce pregnancy complications and preparing them both physically and psychologically for delivery [8, 9]. Moreover, ANC along with other components such as Family Planning, delivery by the skilled birth attendant, and emergency obstetric care provides a comprehensive package to improve maternal and newborn health outcomes [10, 11]. Furthermore, antenatal care service utilization will, in turn, increase the institutional delivery and also encourage women to seek assistance during postnatal period thus reducing maternal morbidity and mortality [10, 11]. Moreover, 3.1 and 3.2 targets of sustainable development goals (SDG) aim to decrease the maternal mortality ratio to less than 70 per 100 000 live births globally and to reduce neonatal mortality to less than 12 per 1000 live births and under-5 mortality to less than 25 per 1000 live births respectively [12]. These two targets of SDGs are supported by multiple global initiatives, indicating the coverage of early ANC services as one of the components to reduce the maternal and perinatal mortality across different countries [11, 13, 14]. ANC coverage has been assessed by different countries through Demographic and Health Surveys (DHS) nationally. According to the global data, approximately 86% of pregnant women seek antenatal care from the skilled birth attendant at least once and only 62% of pregnant women acquire at least four ANC visits [15]. Moreover, in Sub-Saharan Africa and South Asia, only 52 and 46% of pregnant women receive at least four ANC visits respectively [15]. In addition, there are wide inequalities between rural and urban areas regarding the utilization of ANC. This urban-rural gap in coverage of at least four ANC visits exceeds 20 percentage points towards urban areas of South Asia and sub-Saharan Africa [15]. Thus, there are huge differences in antenatal care utilization between urban and rural areas of developing countries. More specifically, DHS surveys have shown that proportion of pregnant women seeking one ANC visit in urban areas is 95.5% in Nepal, 90.7% in India, 89.5% in Bangladesh and 89.1% in Nigeria, while it is 92.1, 72.3, 74.7 and 53% in rural areas of respective countries [16–19]. Likewise, Pakistan demographic health survey (PDHS 2012–2013) also shows that 89.4% of the pregnant women utilize ANC at least once in urban areas as compared to 69.6% in the rural areas, which is a comparatively lower proportion than urban areas of the same country [20]. These figures depict that most of the Pakistani pregnant women in rural areas do not receive antenatal care as compared to their counterparts living in urban areas. Even though Pakistan is facing high maternal and perinatal mortality, there is a dearth of specific research explaining the under-utilization of ANC in rural areas where approximately 63% of the Pakistani population resides [21]. Moreover, with huge expenditures and passage of 22 years, only 33% of the rural Pakistani population is living within access of 5 km (km). Thus in addition to various demographic and socioeconomic factors, lack of access, mainly the road network distance from health care facilities might be a barrier in utilizing the antenatal care services for rural Pakistani women. For instance, studies have shown that general health care utilization for every kind of service is usually affected by distance [22–24]. Furthermore, a “decay effect” of the distance on health care service utilization has been observed, i.e., as the distance increases from the health care facilities ,utilization of services is reduced [22–24]. Access to the facilities also affects the frequencies of services being used [25]. Althoughmultiple factors have been studied affecting the utilization of general health care services, the effect of road network distance particularly on the utilization of ANC has not been assessed in rural Pakistan. Rural areas of Pakistan such as Thatta district are deprived of the facilities providing antenatal care close to the doorsteps of the women. In addition, the road infrastructure in the rural areas is not in the condition to allow women to easily access to the facilities providing ANC services. This situation is further aggravated by the lack of transportation thus further increasing the distance between villages and facilities. However, no scientific research has been carried out to study the effects of distance and transportation on the utilization of ANC services in the rural areas of Pakistan. Therefore, we aimed to carry out this study to assess the association between road network distance from a health care facility and utilization of the ANC among women of reproductive age in Thatta Pakistan. The findings of this study will help in planning and developing strategies for the utilization of ANC for the area under study and will assist policymakers to design suitable strategies for other similar areas across Pakistan.

## Methods

### Study design and study setting

Since 2008, a maternal-newborn health registry (MNHR) has been established by Department of Community Health Sciences Aga Khan University in 14 union councils (UCs) of District Thatta in Sindh province under the Global Network (GN) for Women’s and Children’s Health Research [26, 27]. The MNHR enrolls women during pregnancy and collects data through 6-weeks postpartum to assess pregnancy outcomes in developing countries including Pakistan [26, 27].

From October 2014 to December 2015, we conducted a community- based case-control study in similar 14 UCs of District Thatta Sindh Pakistan, where MNHR is currently functional [26, 27].

### Study population

We defined cases as women who did not utilize the ANC services at all during their last pregnancy, while controls were defined as the women who utilized the ANC services at least once during their last pregnancy. We obtained computer generated identification numbers of cases and controls from the data management system of the MNH registry, which also saves information about ANC utilization in the database [26, 27]. This helped us to identify cases and controls who were approached in the communities for further interviews and data collection. We interviewed both cases and controls from the communities (general population) of the district Thatta after identifying their addresses from the MNH registry. We used a convenience sampling technique to identify cases and controls for the study. Both cases and controls who were residing in 14 UCs of district Thatta, enrolled in the MNH registry and who delivered their babies before the data collection were included in the study. Those women who could not continue their pregnancy due to abortion or who did not provide written informed consent were excluded from the study.

We operationally defined antenatal care as “at least one visit undertaken by a pregnant woman to the health care center providing ANC during her last pregnancy”. The study required a minimum sample of 380 women of reproductive age to achieve the study objective. This sample size was calculated by Open-Epi software and was based on the proportion of exposure related to underutilization of services among controls as 67% [28] and among cases as 80.24% [28], with a power of 80%, the significance level of 5%, and an odds ratio of at least 2. It was found that around 329 women reported utilization of ANC and 51 women were found to be non-utilizers.

### Data collection

The structured questionnaire was developed and translated in the local language, which was used as the main data collection tool to collect data on sociodemographic, fertility-related, and access-related factors. The research staff, familiar with the language and culture of the study setting, administered the questionnaire to the study subjects in the local language. The MNH registry database has also maintained the names and addresses of all the villages and homes of all the women enrolled in the MNHR. This helped data collectors to identify the women who met the eligibility criteria. We provided a list of all eligible women along with their home addresses to the study staff before they approached the women in their homes to schedule an interview with them. The study staff was from the local areas of 14 UCs and had been working in the MNHR for the last five to 8 years. This had helped the staff to establish a relationship with the communities in 14 UCs, which assisted them to conduct interviews for the current study. After assessing the eligibility criteria, the study staff explained the purpose of the study to the women in the local language (Sindhi) before obtaining written informed consent and enrolling them in the study. Since the majority of the women in district Thatta are not adequately educated, therefore, the study staff gave some time to each study participant to discuss the consent with their husbands or mothers-in-law for a better understanding of the study before they participated. In addition, sometimes study participants also took help from the teachers or other educated women in the community to ensure that they could voluntarily participate in the study without any harm.

### Study measures

Upon enrollment, the study staff collected data from the women about all socio-demographic, socioeconomic, fertility-related, and access-related factors by using a structured questionnaire. For example, the study staff collected data regarding woman’s age, woman’s education, husband’s education, place of residence, parity, intention of pregnancy, autonomy of the woman, working status of women, and type of family system. In addition, any formal schooling attended by a woman was considered as literacy. Furthermore, different variables including the occupation of the husband, household income and ownership, construction of the house, household assets, and presence of electricity in the house were used to measure socioeconomic information of the enrolled women. Age of woman was measured in complete years and the data for women’s parity was initially collected as a continuous variable but it was later categorized into nulliparous (having no child), having 1 to 4 children, and multiparous (having at least 5 children). A household was defined as ‘people living together and sharing the same kitchen’ and it was categorized into a nuclear family (single-family) and a joint family (more than one family living in the same household). The data regarding built of the houses were collected and categorized into ‘Pucca houses’ made of concrete, plastered walls and roof and ‘Katcha houses’, whose part or whole was made of wood, clay, and stalks.

This study was approved by the Ethical Review Committee of the Aga Khan University Karachi, Pakistan. In addition, we also got permission from the executive director of the health of district Thatta to carry out this study in 14 UCs of district Thatta. Department of Health is aware of all such studies conducted through the platform of MNHR. Moreover, before approaching study participants, data collectors had to inform community leaders about the research to seek their permission.

### Road network distance measurement with the help of geographic information system

Geographic access has also been found as an important variable in accessing the services. In this particular study, road network distance was measured in kilometers as one of the variables to assess geographic access. Road network distance was measured from women’s homes to the health care centers providing ANC services. Global Positioning System (GPS) coordinates (X (longitude, E)), (Y (latitude, N)) were marked by research study staff using a GPS device to measure the road network distance. The data for GPS coordinates were collected on a separate sheet to record the GPS coordinates either for women’s homes or health care facilities.

Three different sources were used to extract data to measure the distance from women’s homes to health centers providing ANC services. These sources included data on household address from the MNHR in the 14 UCs, GPS coordinates of sampled households and the health care facilities from where women had received ANC services during their last pregnancy and digitized road network of the 14 UCs of Thatta district.

**Fig. 1:**
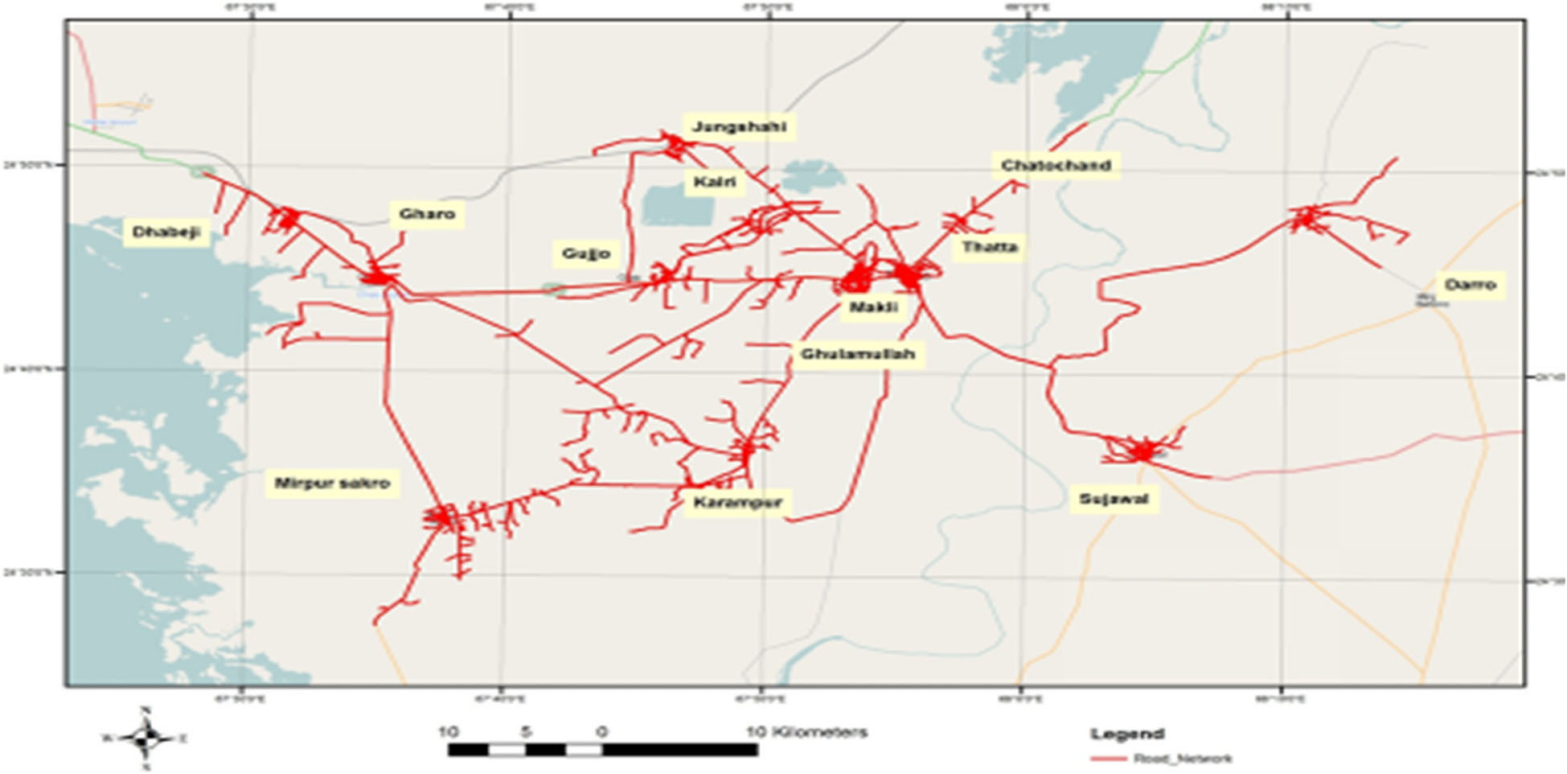
Road network including metal and nonmetal roads in 14 union councils of District Thatta Sindh

**Fig. 2:**
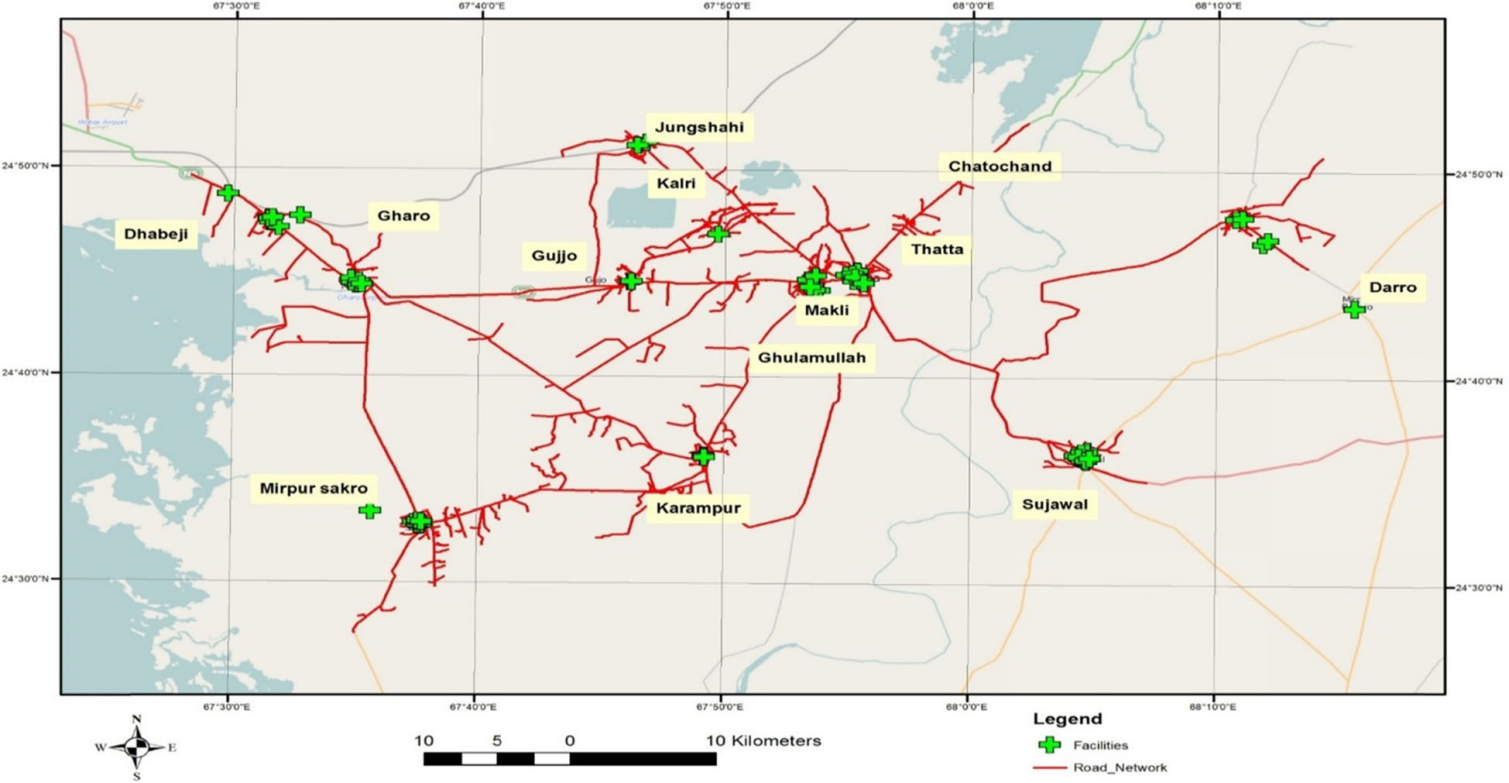
Spatial Distribution of Health facilities providing antenatal care services in 14 union councils of Thatta Sindh

**Fig. 3:**
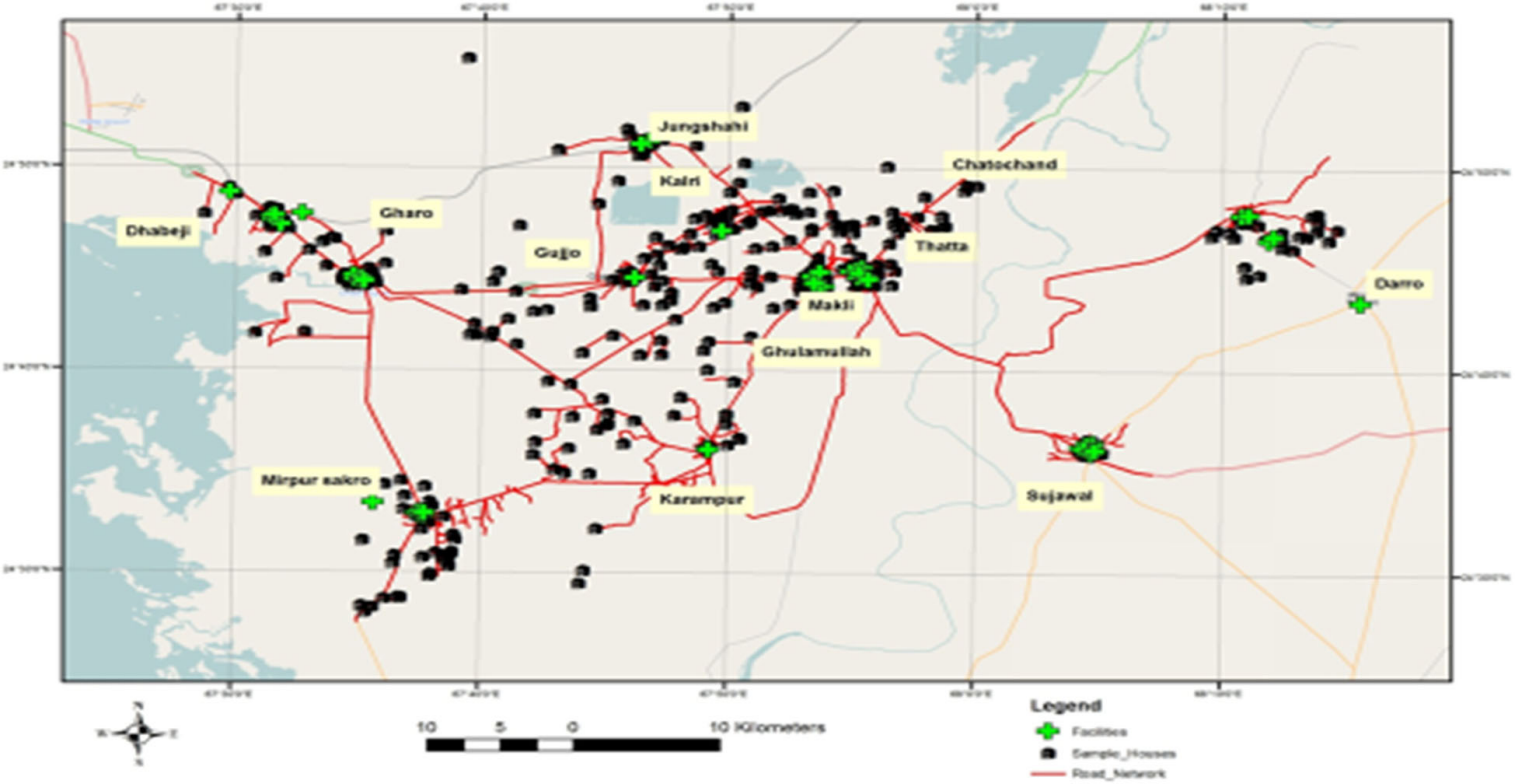
Spatial Distribution of Sampled-Households in 14 union councils of Thatta Sindh

**Fig. 4:**
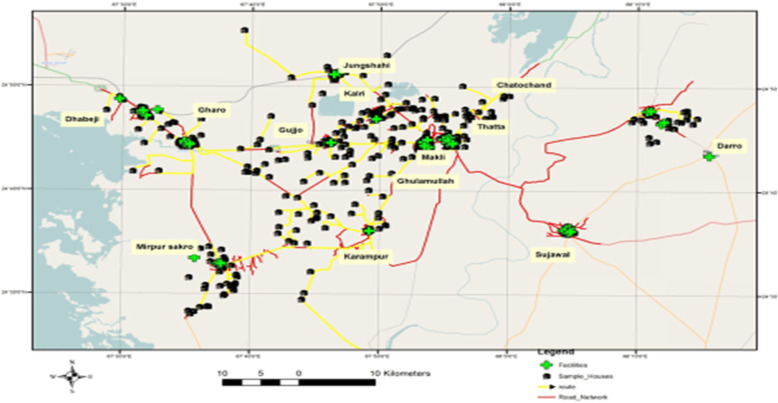
Distance measurement from households to the nearest working family planning center in 14 union councils of district Thatta Sindh. Note: Maps depicted in all figures are my own generated using GIS software.

High-resolution 0.6 m quick-bird satellite imagery was acquired from Google Earth Pro to establish a geographic information system (GIS) database of the transportation system in 14 UCs of the Thatta district. Satellite imagery plays a vital role in raster data handling, a type of GIS data, by providing an excellent basis to extract the pertinent information. The images of 14 UCs were downloaded in numerous phases by mosaicking individual images to get a single combined image of each UC that could be used for geo-rectification, or confirmation of geographic location to reference the individual centers by their exact pixel addresses [29]. Thatta district’s road network comprises of the national highway, concreted, and non-paved roads (Fig. 1). The ArcGIS software was used to develop the GIS-based digitized road map of 14 UCs. This was followed by a GPS-based survey in the14 UCs to define the exact location of health centers providing antenatal care services. The (X (longitude, E)), (Y (latitude, N)) coordinates of health care centers such as private facilities, houses of Lady Health Workers (LHW), and government facilities were marked using a GPS device and these locations were integrated with GIS software as shown in Fig. 2. Thatta district is classified into rural and urban regions due to the complexity of the transportation system and infrastructure. GPS coordinates were marked in front of sampled households of interest in the urban regions, while the center of the village was used to mark the coordinates in the rural areas, as shown in Fig. 3. The data collected through GPS survey was imported into Arc GIS version 9.3 to measure the road network distance from participant’s homes to health centers providing ANC services. The road network distance was measured from household to the health center where a woman had gone to receive antenatal care during her last pregnancy as shown in Fig. 4.

### Statistical analysis

Chi-square and independent t-tests were applied for comparative analysis between utilizers and non-utilizers for categorical and continuous variables respectively. Logistic regression was done to identify variables, which could predict the antenatal care utilization. Multi-collinearity was assessed and biological plausible interactions were assessed between independent variables. Finally, variables were incorporated in the final model by evaluating their significance at a *p*-value of less than 0.05. The magnitude of the effect of the independent variables associated with the utilization of antenatal care was assessed through adjusted odds ratios (AOR) and the significance of association was assessed through the corresponding 95% CI. Statistical Package of Social Sciences (SPSS) version 19.0 was used for data analysis.

## Results

A total of 380 participants were interviewed and analyzed in this study. The mean age of the participants was 28 years (SD 5.65 years), while the mean parity of the participants was 3.5 (SD 2.6). The majority of the participants (81.3%) did not have formal schooling and about one-quarter (26.3%) were found to be working women. Approximately 90% of the participants’ owned a house and half of the study participants’ were living in Katcha house. The average size of the household was 10.33 persons (SD 7.01). Around 62% of the participants were living in a joint family with their in-laws and other relatives. More than two-thirds of the participants (68%) had the facility of electricity in their homes, while only 9.3% of the participants reported owning any type of vehicle.

Table 1 represents the distribution of various socio-demographic and reproductive factors with respect to the utilization of ANC. It shows that around 36.8% of the women aged less than equal to 25 years were found to be ANC utilizers, while 37.3% of the same age group was found to be non-utilizers of ANC. Similarly, 37.3% of the non-utilizers reported having at least 5 children and 28.6% of the utilizers reported to have the same number of children. Regarding the education level of participants, 20.7% of utilizers and 5.9% of the non-utilizers were found to be literate. Similarly, regarding the husband’s education, 44.4% of husbands of utilizers and 23.5% of husbands of non-utilizers were found to be literate. Regarding the current working status of the woman, 24.0% of utilizers and 41.2% of non-utilizers reported that they were currently working for earning purposes.

**Table 1:** Socio-demographic characteristics of women utilizing antenatal care services in District Thatta, Crude Odds Ratios and 95%CI (*n* = 380)

Characteristics	Not utilizers of ANC services (***n*** = 51) n (%)’	utilizers of ANC services (***n*** = 329) n (%)	Odds Ratio	95%CI	***P***-value
**Age of the woman at the time of interview**					
≤ 25 years	19 (37.3)	121(36.8)	1		
More than 25 years	32(62.7)	208(63.2)	1.02	0.55–1.88	0.95
**Parity**					
Multiparous	19(37.3)	94(28.6)	1		
1 to 4 children	28 (54.9)	203(61.7)	1.46	0.78–2.75	
Nulliparous	4(7.8)	32(9.7)	1.62	0.51–5.11	0.45
**Educational Status of women**					
Illiterate	48 (94.1)	261(79.3)	1		
Literate	3 (5.9)	68(20.7)	4.17	1.26–13.79	0.02
**Educational Status of husband**					
Illiterate	39(76.5)	183(55.6)	1		
Literate	12(23.5)	146(44.4)	2.59	1.31–5.13	0.006
**Current working status of woman**					
Yes	21(41.2)	79(24.0)	1		
No	30(58.8)	250(76.0)	2.21	1.20–4.09	0.01
**Current working status of husband**					
Yes	50(98.0)	313(95.1)	1		
No	1 (2.0)	16(4.9)	2.55	0.32–19.7	0.37
**Woman’s autonomy**					
Autonomy	44 (86.3)	262(79.6)	1		
No Autonomy	7(13.7)	67(20.4)	1.61	0.69–3.73	0.27
**Place of residence**					
Rural	46(90.2)	236(71.7)	1		
Urban	5(9.8)	93(28.3)	3.62	1.39–9.41	0.008
**Type of Family System**					
Nuclear	21(41.2)	123(37.4)	1		
Joint	30(58.8)	206(62.6)	1.17	0.64–2.14	0.60
**Having Electricity**					
No	32(62.7)	88(26.7)	1		
Yes	19 (37.3)	241(73.3)	4.61	2.48–8.66	< 0.001
**Type of House**					
Katcha	40(78.4)	156(47.4)	1		
Pakka	11(21.6)	173(52.6)	4.03	2.00–8.13	< 0.001
**Intention of pregnancy**					
Wanted	35(68.6)	106(32.2)	1		
Unwanted	16(32.6)	223(67.8)	1.04	0.55–1.96	0.90
**Knowledge about the antenatal care**					
No	30(58.8)	64(81.4)	1		
Yes	21(41.2)	265(80.5)	5.91	3.18–11.01	0.90

With respect to the place of residence, 71.7% of the utilizers and 90.2% of the non-utilizers were living in the rural (remote villages) areas, while 28.3% of the utilizers and 9.8% of the non-utilizers were living in the urban (city) areas. As far as a facility of electricity was concerned, 73.3% of the ANC utilizers and 37.3% of the non-utilizers reported having the facility of electricity in their homes, while 26.7% of the utilizers and 62.7% of the non-utilizers did not have the facility of electricity. Regarding the type of house, 52.6% of the utilizers were living in the Pakka houses as compared to 21.6% of the non-utilizers. With respect to the knowledge of ANC, 80.5% of the utilizers and 41.2% of the non-utilizers had knowledge of ANC.

Table 2 shows the access-related factors affecting the utilization of Antenatal Care. Regarding the road network distance calculated with GIS in kilometers, 21.3% of the utilizers and 15.7% of the non-utilizers were living within 5 km of the radius of the facilities providing antenatal services. Around 10.0% of the utilizers and 3.9% of the non-utilizers reported having any type of transportation. Moreover, 59.9% of utilizers and 41.2% of the non-utilizers reported having paved roads near their houses as shown in Table 2.

**Table 2:** Access to Antenatal care services among married women of District Thatta , Crude Odds Ratios and 95%CI (*n* = 380)

Characteristics	Not utilizers of ANC services (***n*** = 51) n (%)	utilizers of ANC services (***n*** = 329) n (%)	Odds Ratio	95%CI	P-value
**Road network distance**					
≥ 5 km	43(84.3)	259 (78.7)	1		
< 5 km	8(15.7)	70(21.3%)	1.45	0.65–3.23	0.36
**Perceived distance by respondent between house and Facility**					
At walking distance	12(23.5)	61(18.5)	1		
Far/Very Far	39(76.5)	268(81.5)	1.35	0.67–2.73	0.40
**Availability of Personal transportation**					
No	49(96.1)	296(90.0)	1		
Yes	2 (3.9)	33(10.0)	2.73	0.64–11.75	0.18
**Types of Roads**					
Paved	21(41.2)	197(59.9)	1		
Non-Paved	30(58.8)	132(40.1)	2.13	1.17–3.88	0.01
**Mode of conveyance used by respondent to visit the Facility**					
Own /Hired conveyance	43(84.3)	252(76.6)	1		
Walking/Public transport	8(15.7)	77 (23.4)	1.64	0.74–3.64	0.22

Tables 1 and 2 also shows univariate analysis with crude odds and 95% confidence intervals (CIs). It was found that literate women were 4.17 times likely to utilize antenatal care services as compared to the illiterate women [OR. 4.17; 95% CI (1.26–13.79)]. Similarly, women whose husbands were literate were 2.59 times likely to utilize ANC services as compared to their counterparts [OR. 2.59; 95% CI (1.31–5.13)]. Women who were not working currently for earning were 2.21 times likely to utilize antenatal care as compared to women who were currently working for earning [OR. 2.21; 95% CI (1.20–4.09)]. Women who were living in urban areas were 3.62 times likely to utilize antenatal care services as compared to women who were living in rural areas [OR: 3.62; 95% CI (1.39–9.41)]. Women having the facility of electricity were 4.61 times likely to utilize the ANC services as compared to those who did not have the facility of electricity [OR: 4.61; 95% CI (2.48–8.66)]. Women who were living in Pakka house were 4.03 times likely to utilize the ANC services as compared to those who were living in Katcha house [OR: 4.03; 95% CI (2.00–8.13)].

With respect to the access-related factors, it was found that women living at less than 5 km from the health care facility were 1.45 times likely to utilize ANC services [OR: 1.45; 95% CI (0.65–3.23)] as compared to the women living farther away at more than 5 km distance from health care facilities. However, the results for this association were not found to be significant. Interestingly, those who reported having non-paved roads near to their homes were 2.13 times likely to utilize ANC as compared to the ones who reported having paved roads [OR: 2.13; 95% CI (1.17–3.88)].

In multivariate analysis, it was found that women living at a shorter distance of less than 5 km were 1.21 times likely to utilize ANC services [Adjusted OR. 1.21; 95% CI (0.49–2.99)] as compared to the women living at more than 5 km distance from health care facilities while adjusting for other variables in the model. However, the results were not statistically significant and there was a significant association found between road network distance and utilization of ANC services. With respect to the parity, we found that nulliparous women were 4.10 times likely to utilize antenatal care as compared to multiparous women while adjusting for other factors [Adjusted OR. 4.10; 95% CI (1.10–15.26)] (Table 3). Similarly, women who had knowledge of antennal care were 6.60 times likely to utilize ANC services as compared to those who had no knowledge of antenatal care [Adjusted OR. 6.60; 95% CI (3.33–13.05)]. Women who had the facility of electricity were 3.11 times likely to utilize the ANC services [Adjusted OR.; 3.11 95% CI (1.51–6.41)] as compared to their counterparts. Those women who were living in Pakka house were also 2.58 times likely to utilize the ANC services as compared to those who were living in Katcha house while adjusting for other variables [Adjusted OR: 2.58; 95% CI (1.15–5.82)] as shown in Table 3.

**Table 3:** Multivariate analysis for factors associated with utilization of antenatal care services among married women of District Thatta Sindh, Adjusted Odds ratios (95% CI) (*n* = 380)

Characteristics	Adjusted Odds Ratio	95%CI
**Road network distance**		
≥ 5 km	1	
< 5 km	1.21	0.49–2.99
**Parity**		
Multiparous	1	
1 to 4 children	1.49	0.74–3.05
Nulliparous	4.10	1.10–15.26
**Knowledge about the antenatal care**		
No	1	
Yes	6.60	3.33–13. 05
**Having Electricity**		
No	1	
Yes	3.11	1.51–6.41
**Type of House**		
Katcha	1	
Pakka	2.58	1.15–5.82

## Discussion

This study was one of the few attempts towards an assessment of the effect of road network distance on antenatal care utilization among married women of reproductive age in Thatta Sindh Pakistan. Our study indicates that road network distance has no measurable impact on antenatal care utilization among married women in the catchment population of Thatta district Pakistan. This association between road network distance and ANC utilization was also assessed after categorizing the distance into > 2, > 5 and > 10 km but the association remained insignificant.

This might be because healthcare centers providing ANC services are not very far from the homes of women as evidenced by geographical data in this study. However, our study found that nulliparous women knowing of antenatal care, living in Pakka house with the facility of electricity were found to utilize the ANC services in the study area.

The findings regarding the utilization of ANC by nulliparous women can be explained by the fact that due to the first baby, nulliparous women might be more mindful about the health of their upcoming baby. This premise is consistent with other studies being carried out across the world which suggests that parity is inversely related to ANC utilization [30–33]. This finding can be explained by the fact that nulliparous women might be less experienced as compared to the multiparous women thus willing to utilize ANC more as compared to the multiparous women and this is also supported by the literature [34]. Moreover, nulliparous women might be less confident and more conscious for their upcoming baby, which might encourage them to utilize ANC services more than multiparous women [34]. In addition to this, the high utilization of ANC among nulliparous women could be because of time management, more resources in the family, and positive perceptions about the benefits of ANC [35]. Hence parity influences the utilization of ANC and as parity increases, the experience of timely initiation of ANC decreases as these women might give less value to the ANC services as compared to nulliparous women [36]. This finding is supported by one of the studies conducted in Tanzania, where nulliparous were about two times more likely to book early for ANC services as compared to the women with more children [37].

In addition to this, we found that women knowing of antenatal care were more likely to utilize antenatal care services as compared to the ones who did not know about ANC services. Having knowledge about health enables women to be aware of their health status in order to seek appropriate health services in a timely manner [38]. Moreover, the literature reveals that adequate knowledge provides benefits of antenatal care and women become aware of the complications associated with pregnancy. For example, one of the studies conducted in Ethiopia found that the women who had a better knowledge of antenatal care were 3.54 times likely to utilize the services than those who did not have any knowledge [39]. Likewise, a study conducted in urban areas of Pakistan found that women having knowledge about ANC are more likely to utilize ANC services [40].

In addition, our study demonstrated that women who had the facility of electricity at their homes were more likely to utilize ANC services as compared to those who did not have the facility of electricity. These findings are consistent with one of the studies conducted in the rural area of North Sindh Pakistan, where women living in houses with the facility of electricity were found to utilize antenatal care more as compared to their counterparts [21]. The possible explanation of this finding is that the presence of electricity in a household of a rural setting might be an indirect measure of accessibility to services and it might be a sign of a better social class. This is further supported by literature, which highlights that a better social class has been found to be associated with better utilization of ANC among women of reproductive age [41, 42].

We also assessed the construction of the house as one of the indicators of the socio-economic status of women. These findings regarding the construction of the house are also consistent with the study conducted in other parts of Pakistan [21]. This depicts that women who were living in well-constructed houses (Pakka) were utilizing antenatal care more compared to those living in poorly constructed houses (Katcha). Living in well-constructed houses is also a reflection of good socioeconomic status and this is also supported by the literature showing that women with better socioeconomic status are more likely to avail ANC services [43–45]. We did not perform analysis on ownership of the house because almost 100% of the residents were owners of their houses in the study area.

### Strengths and limitations of the study

This study has certain potential strengths. The principal strength of this study is that to the best of our knowledge this is the first epidemiological study in the rural area of Pakistan measuring the road network distance by using GIS technology mainly for investigating the association with ANC utilization. Unlike other studies, we measured road network distance rather than straight-line distance with the help of GIS by doing a proper road network analysis. Moreover, we calculated the distance from that facility where women had gone to avail the ANC services rather than measuring the distance from the nearest facility. This road network distance is a significantly better representation of access and provides more accurate estimates than the simple crow-fly distance. Since the variable of distance was calculated objectively and prospectively, therefore, chances of recall bias regarding this variable are minimized. In addition, we used a validated questionnaire to assess the determinants of ANC utilization in rural Thatta Pakistan. Moreover, in contrast to the hospital-based studies, this study used a community-based approach to recruit participants from individual households, therefore, the results of this study can be generalized to rural areas of Pakistan and other similar areas of neighbouring countries.

Attempts were made to conduct this study in the best possible way, however, as with any study there are certain limitations of the study. First, because of limited resources, the random selection of the participants could not be done and participants were recruited conveniently. Second, there is the possibility of recall bias regarding some of the variables such as antenatal care services utilization, however, this should not affect the results much because most of the variables did not require participants to recall. Secondly, travel time could not be calculated, as it is a more accurate indicator of time and energy expended by women traveling to the facility for availing ANC services. Since the average speed of each mode of transportation was not calculated and information about the mode of conveyance used by the women to visit the health centers was also incomplete, therefore, travel time could not be calculated. Besides this, there are numerous other factors such as road type, topology, the day of time, traffic congestion, urban or rural road, land use, and season which could affect road travel time [46] and all these factors require a lot of time and labor force to calculate travel time. In addition, we did not assess certain determinants such as the provider’s attitude towards the client, waiting time at the facility and travel time from the facility, which is also an important factor of any service utilization generally.

## Conclusion

In summary, the study informed that road network distance has no measurable impact on antenatal care utilization among married women in the catchment population of Thatta district Pakistan. However, this study revealed that nulliparous women having knowledge of antenatal care living in well-constructed houses equipped with electricity were found to be utilizers of antenatal care services in the rural area of Pakistan. Thus, the study recommends that nulliparous women should be given more knowledge and awareness to improve the ANC utilization and socioeconomic status to increase the utilization in rural areas of Pakistan. Given the findings of the study, it is recommended that awareness and health education sessions should be arranged for pregnant women to increase their knowledge about antenatal care services. These women should be given awareness about new guidelines of ANC services, their benefits and the location from where the services can be sought. In addition, the socioeconomic status of the women needs to be improved so that they can be enabled to afford the ANC services from the health care facilities. Future studies are required to assess the determinants at individual, household, community and facility levels to understand the determinants of ANC services holistically.

## Data Availability

All data generated or analyzed during this current study available from the corresponding author on reasonable request.

## Abbreviations

ANC: Antenatal Care
AKU: Aga Khan University
CHS: Community Health Sciences
CI: Confidence Interval
ERC: Ethical review committee
GA: Gestational age
GIS: Geographical information system
GPS: Global Positioning System
GN: Global Network
ID: identification numbers
KM: kilometer
LHW: Lady Health Worker
MNHR: Maternal and newborn Health Registry
NGOs: Non- Governmental organizations
OR: Odds Ratio
SD: Standard Deviation
SDG: Sustainable Development Goals
UC: Union council
WHO: World Health Organization
